# Upward trends in new, rifampicin-resistant and concurrent extrapulmonary tuberculosis cases in northern Guizhou Province of China

**DOI:** 10.1038/s41598-021-97595-8

**Published:** 2021-09-09

**Authors:** Ling Chen, Xuefeng Fu, Peng Tian, Qing Li, Dan Lei, Zhangli Peng, Quanxian Liu, Nana Li, Jianyong Zhang, Peng Xu, Hong Zhang

**Affiliations:** 1 Tuberculosis Division of Respiratory and Critical Care Medicine, Affiliated Hospital of Zunyi Medical University, Zunyi, 563003 China; 2 Institute of Life Sciences, Zunyi Medical University, Zunyi, 563003 China; 3 Z-BioMed, Inc., Rockville, MD 20855 USA

**Keywords:** Antimicrobials, Bacteria, Tuberculosis

## Abstract

Similar to global trends, the incidence rate of tuberculosis (TB) in China declined from 2000 to 2018. In this study, we aimed to evaluate TB trends in northern Guizhou Province and identify risk factors associated with rifampicin-resistant (RR) and concurrent extrapulmonary TB (EPTB). We analyzed data of TB patients hospitalized in Affiliated Hospital of Zunyi Medical University from 2011 to 2018, and assessed correlations between demographic characteristics of patients and RR-TB as well as concurrent EPTB. Our results showed that numbers of new, retreated, RR-TB and concurrent EPTB cases increased gradually from 2011 to 2018. Retreated patients had the highest odds of RR-TB but a lower likelihood of concurrent EPTB compared to new patients. Patients between 21 and 40 years of age had a higher likelihood of RR-TB compared to those 20 years and younger. Female patients and patients from Bijie city as well as the Miao ethnic minority had higher odds of concurrent EPTB. In summary, our data demonstrate upward trends in new, rifampicin-resistant and concurrent extrapulmonary TB cases in northern Guizhou Province of China, which should not be overlooked especially during and post the COVID-19 pandemic because TB is a greater long-term global health threat than COVID-19.

## Introduction

As the leading infectious disease of the world, tuberculosis (TB) caused an estimated 1.2 million deaths among HIV-negative people globally in 2018^1^, more than the deaths caused by annual influenza epidemics (290,000 to 650,000) estimated by the World Health Organization (WHO) and the 2009 H1N1 influenza pandemic (151,700 to 575,400) estimated by the Centers for Disease Control and Prevention (https://www.cdc.gov/flu/pandemic-resources/2009-h1n1-pandemic.html). TB and malaria are considered to be greater long-term global health threats than the COVID-19 pandemic^2^. Among 7.25 million incident cases of TB reported to WHO in 2018, 15% were extrapulmonary TB (EPTB) and 2.6% were multidrug-resistant/rifampicin-resistant TB (MDR/RR-TB)^1^. Although global trends in the estimated number of all TB cases and the incidence rate continued to decline from 2000 to 2018, six of the 30 high TB burden countries (Bangladesh, Central African Republic, DPR Korea, DR Congo, Nigeria, and Papua New Guinea) showed no changes and two countries (Liberia and Mozambique) showed slightly upward trends in TB incidence rates during the same period^1^. Downward trends were also reported in overall TB incidence rates in developed countries such as Japan from 1997 to 2016^3^, Canada and the USA from 1997 to 2015^4^; and in the MDR/RR-TB rate in foreign-born persons in Italy from 2009 to 2016^5^.

**Figure 1:**
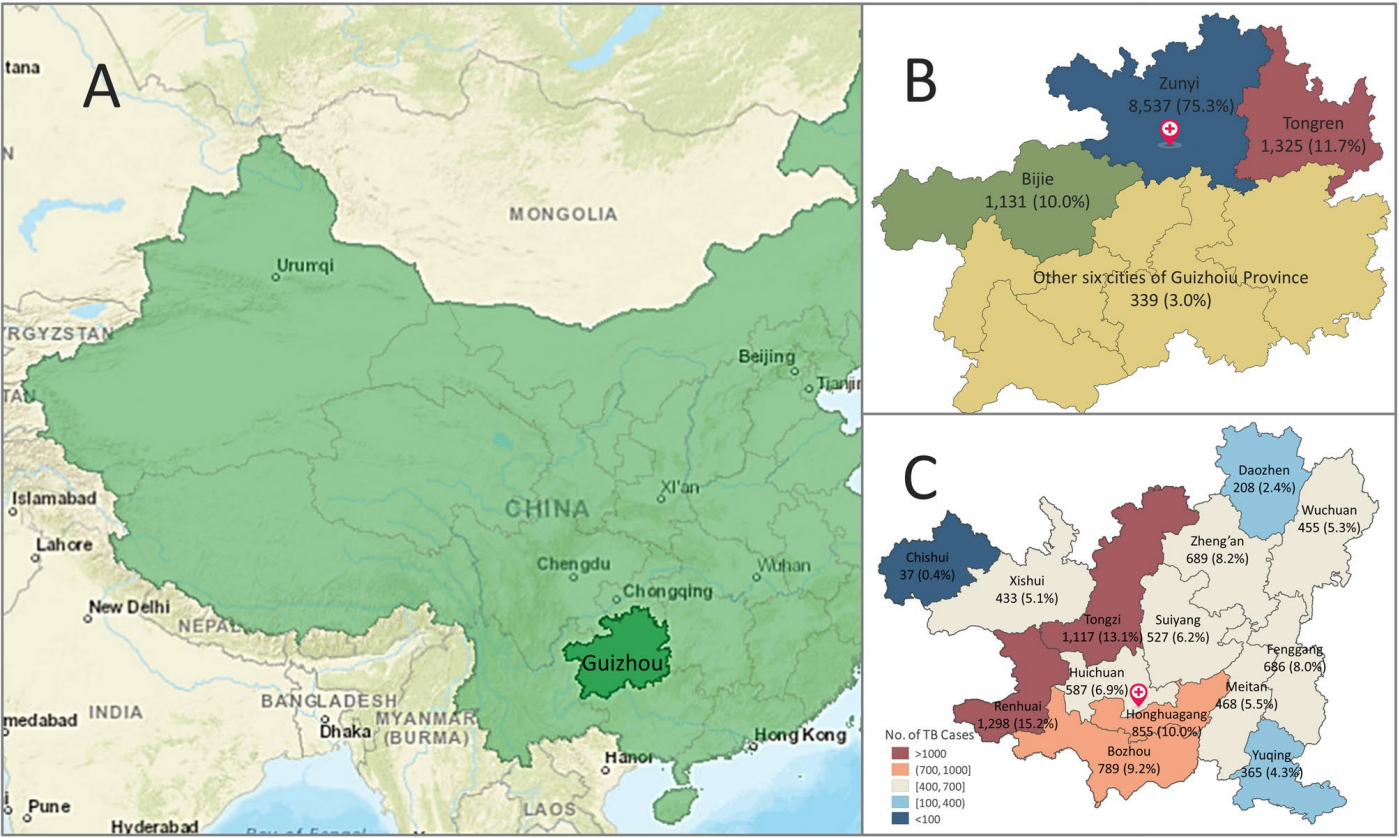
Geographical locations of Guizhou Province of China and its prefectural cities. (**A**) The geographical location of Guizhou Province (dark green color) of China (light green color). (**B**) The geographical locations of three prefectural cities (Bijie, Tongren, and Zunyi) and other six cities or autonomous prefectures of Guizhou Province. (**C**) Number and percentage of TB cases in different administrative units of Zunyi city. The location of Affiliated Hospital of Zunyi Medical University is indicated with a red cross. The maps were created using GeoDa software (Version 1.14) downloaded from the GeoDa website (https://geodacenter.github.io). The map data of Guizhou Province of China and cities in Guizhou were from the GADM website (https://gadm.org/).

China is one of the high TB/MDR-TB burden countries and the TB epidemic status mainly depends on regional geographic, socioeconomic, and cultural variations. The total TB incidence rate in China continuously declined from > 100/100,000 in 2000 to 61/100,000 population in 2018^1^, while similar declines were also observed in many provinces and cities of China^6–10^. However, the TB incidence rate in Guizhou (133.5/100,000 population), a relatively underdeveloped southwestern province of China (Fig. 1A), was approximately two times higher than the national average (63.4/100,000) and ranked third following Xinjiang (184.5/100,000) and Tibet (140.2/100,000) Autonomous Regions in 2015^11^. The prevalence of drug-resistant TB (27.5%) and MDR-TB (10.9%) in Guizhou Province was also higher than the average numbers in China and in the world^12^. Analysis of 8 years of data from four provinces in western China showed that the overall incidence rate of pulmonary TB (PTB) in ethnic minority areas increased gradually from 114.4/100,000 in 2011 to 140.9/100,000 in 2018^13^. Our previous study indicated that the trends in drug-resistant and MDR-TB declined at a major hospital in Guizhou Province from 2008 to 2015^14^.

In order to enhance control strategies and improve treatment outcomes for RR-TB and EPTB, it is important to evaluate TB epidemic characteristics and trends in RR-TB and concurrent EPTB in high TB burden areas of Guizhou Province. The objectives of this study were to extend our previous study by analyzing data collected from 2011 to 2018 about inpatient TB cases at the same hospital; to evaluate the current TB trends and geographic variations in northern Guizhou Province of China; and to identify risk factors associated with RR-TB and concurrent EPTB.

## Methods

### Ethics approval

This observational retrospective study was approved by the Ethics Committee of Affiliated Hospital of Zunyi Medical University, China. Because diagnostic tests were routinely used in clinical practice and all individual patient information was removed before analysis, the Ethics Committee of Affiliated Hospital of Zunyi Medical University waived the requirement of informed consent from individual patients.

### Data sources and procedures

This study was carried out at the Affiliated Hospital of Zunyi Medical University, which is a tertiary general hospital and one of the major medical centers within the healthcare system of Guizhou Province. This hospital was designated by the provincial government in 2014 as a main part of a regional referral system specialized in the treatment of MDR-TB patients diagnosed at the hospital and those referred from primary/secondary level facilities in northern Guizhou Province, which consists of three prefectural cities (Bijie, Tongren, and Zunyi) (Fig. 1B).

Demographic and clinical data of patients admitted to the TB Division of the hospital were collected and recorded from January 1, 2011 to December 31, 2018. The collected data were stored in the data center of our hospital, which included patient information (gender, age, ethnicity, and address), treatment history, PTB, concurrent EPTB, and drug susceptibility testing (DST) results. To ensure the data used in this study were anonymous, any information related to patient identity was filtered under the supervision of the data center manager. Our collected addresses contained only names of cities or counties where patients lived, consequently, there was no way to find out patient identity without road names and street addresses. The criteria used to define active TB cases included clinical features, imaging results, and laboratory tests according to the health industry standards of China (WS 288–2008 and WS 288–2017). Diseases caused by nontuberculosis mycobacteria (NTM) were excluded from this study. To avoid duplication of cases, each patient was assigned a unique hospital admission number which was linked to patient’s National Identification Number or Social Security Number and used to organize all data including clinical records. We were not authorized to access the identity information of patients which was strictly controlled by the hospital and local governments.

Clinical isolates of *M. tuberculosis* (*M. tb*) were collected from sputum samples of TB or suspected TB patients as part of routine hospital laboratory procedures, and only one isolate per patient was collected to avoid duplication. Processing and inoculation of collected sputum specimens were performed at the Laboratory of Respiratory Medicine of the hospital by following the procedures recommended by WHO. The Laboratory was certified by the China CDC to conduct DST on TB isolates, and DST procedures were described previously^14,15^. Four methods for *M. tb* detection and two methods for rifampicin (RIF) susceptibility testing were conducted in this study. GeneXpert MTB/RIF (Xpert) (Cepheid Inc.; Sunnyvale, CA, USA) and Loopamp MTBC Detection Kit (TB-LAMP) (Eiken Chemical Co.; Tokyo, Japan) were used in accordance with the manufacturer’s instructions. All methods used in this study were performed in accordance with the relevant guidelines and regulations.

### Statistical analysis

We used Stata version 16.0 (StataCorp, College Station, Texas, USA) for statistical analyses of linear regression, univariate and multivariate logistic regression, and confidence intervals (CI). The trends of new, retreated, PTB, PTB concurrent with EPTB, rifampicin-susceptible and -resistant TB cases were analyzed by chi-square test. We created maps and annotated geographic and clinical data in those maps using GeoDa software (Version 1.14) downloaded from the GeoDa website (https://geodacenter.github.io). The map data of Guizhou Province of China and cities in Guizhou were from the GADM website (https://gadm.org/), while the map of administrative units in Zunyi was modified according to the current administrative divisions.

## Results

Among 11,610 TB cases collected from 2011 to 2018, 150 (1.3%) EPTB-only cases were excluded from this study because they were not common and the risk of transmission by EPTB-only patients was much lower than patients with PTB and PTB concurrent with EPTB. We analyzed 11,460 PTB cases, of which 98.9% (11,334) were from Guizhou Province, and 1.0% (116) from other provinces of China. The geographical locations of Guizhou Province and its prefectural cities were shown in Fig. 1. Among TB cases from Guizhou, 10,993 (97%) were from northern Guizhou, 8537 from Zunyi, 1325 from Tongren, and 1131 from Bijie (Fig. 1B, Table 1).

**Table 1:** Univariate logistic regression analysis of characteristics of TB patients in association with treatment history and TB type.

Characteristics	New, n (%)	Retreated, n (%)	OR (95% CI)	*P* value	PTB, n (%)	PTB & EPTB, n (%)	OR (95% CI)	*P* value	Total
**Gender**									
Male	5526 (78.8)	1486 (21.2)	1.00	N/A	4377 (62.4)	2635 (37.6)	1.00	N/A	7012
Female	3654 (82.1)	794 (17.9)	0.81 (0.73–0.89)	< 0.0001	2511 (56.5)	1937 (43.5)	1.28 (1.19–1.38)	< 0.001	4448
**Age (years)**									
≤ 20	1398 (92.3)	117 (7.7)	1.00	N/A	775 (51.2)	740 (48.8)	1.00	N/A	1515
21–40	2429 (82.0)	535 (18.0)	2.63 (2.13–3.25)	< 0.0001	1849 (62.4)	1115 (37.6)	0.63 (0.56–0.72)	< 0.0001	2964
41–60	2528 (74.6)	861 (25.4)	4.07 (3.32–4.99)	< 0.0001	2164 (63.9)	1225 (36.1)	0.59 (0.52–0.67)	< 0.0001	3389
> 60	2825 (78.6)	767 (21.4)	3.24 (2.64–3.98)	< 0.0001	2100 (58.5)	1492 (41.5)	0.74 (0.66–0.84)	< 0.0001	3592
**Ethnicity**									
Han	8172 (80.2)	2015 (19.8)	1.00	N/A	6151 (60.4)	4036 (39.6)	1.00	N/A	10,187
Tujia	395 (77.5)	115 (22.5)	1.18 (0.95–1.46)	0.127	313 (61.4)	197 (38.6)	0.96 (0.80–1.15)	0.655	510
Miao	265 (79.3)	69 (20.7)	1.06 (0.81–1.38)	0.692	169 (50.6)	165 (49.4)	1.49 (1.20–1.85)	< 0.0001	334
Kelao	221 (83.4)	44 (16.6)	0.81 (0.58–1.12)	0.202	164 (61.9)	101 (38.1)	0.94 (0.73–1.21)	0.621	265
Others	127 (77.4)	37 (22.6)	1.18 (0.82–1.71)	0.376	91 (55.5)	73 (44.5)	1.22 (0.90–1.67)	0.205	164
**City (n = 11,448)**									
Zunyi	6887 (80.7)	1650 (19.3)	1.00	N/A	5305 (62.1)	3232 (37.9)	1.00	N/A	8537
Tongren	1014 (76.5)	311 (23.5)	1.28 (1.12–1.47)	< 0.0001	733 (55.3)	592 (44.7)	1.33 (1.18–1.49)	< 0.0001	1325
Bijie	910 (80.5)	221 (19.5)	1.01 (0.87–1.19)	0.865	593 (52.4)	538 (47.6)	1.49 (1.31–1.69)	< 0.0001	1131
Other GZ cities	264 (77.9)	75 (22.1)	1.19 (0.91–1.54)	0.202	181 (53.4)	158 (46.6)	1.43 (1.15–1.78)	0.001	339
Outside GZ	95 (81.9)	21 (18.1)	0.92 (0.57–1.48)	0.740	65 (56.0)	51 (44.0)	1.29 (0.89–1.86)	0.179	116
**Treatment history**									
New	N/A	N/A	N/A	N/A	5367 (58.5)	3813 (41.5)	1.00	N/A	9180
Retreated	N/A	N/A	N/A	N/A	1521 (66.7)	759 (33.3)	0.70 (0.64–0.77)	< 0.0001	2280
Total	9180 (80.1)	2280 (19.9)	N/A	N/A	6888 (60.1)	4572 (39.9)	N/A	N/A	11,460

*CI* confidence interval, *EPTB* extrapulmonary TB, *GZ* Guizhou province, *N/A* not applicable, *OR* odds ratio, *PTB* pulmonary TB, *PTB & EPTB* pulmonary TB concurrent with EPTB.

Overall, 7012 male (61.2%) and 4448 female (38.8%) TB patients were diagnosed, and 21.2% of male patients and 17.9% of female patients were retreated. Female patients had lower odds of being a retreated TB case (Odds ratio (OR) of 0.81) but higher odds of being a concurrent EPTB case (OR 1.28) than male patients. The population of Guizhou Province is composed of Han ethnicity, which makes up the majority of the population, and many ethnic minorities. In this study, the Han majority accounted for 88.9% of TB patients, while 17 ethnic minorities accounted for 11.1% of TB patients (Table 1). The top three ethnic minority groups were Tujia, Miao, and Kelao.

The majority of TB patients were over 18 years of age (94%), and 6% of them were between 11 and 17 (Median 48 years, range 11–99). Compared to those 20 years and younger, patients in the 41–60 age group had the highest odds of having retreated TB (OR 4.07) and the lowest odds of having concurrent EPTB (OR 0.59). Patients from the Miao ethnic group had the highest likelihood of having EPTB (OR 1.49, *P* < 0.0001) in comparison with the Han majority. Additionally, patients from Bijie had the highest odds of having EPTB (OR 1.49, *P* < 0.0001) compared to those from Zunyi (Table 1).

We have used Xpert assay to simultaneously detect *M. tuberculosis* complex (MTBC) and RIF resistance since 2016, and have used TB-LAMP to detect MTBC since 2017. At least one of the four detection methods was applied to 98.5% (11,286/11,460) of cases with positive rates of 25.6% for AFB smear, 39.4% for bacterial culture, and 48.4% for Xpert. The percentage of positive PTB cases detected by any of the four methods was 35.4%. During the study period, the proportion of confirmed *M. tb* cases increased from 21.3% in 2012 to 34.7% in 2016. With the addition of Xpert and TB-LAMP, this proportion increased to over 49% in 2017 and 2018. Overall, 15.7% of tested cases for RIF were RR-TB (Table 2).

**Table 2:** Univariate and multivariate logistic regression analyses of characteristics of TB patients in association with rifampicin resistance.

Characteristics	RS-TB, n (%)	RR-TB, n (%)	Total	OR (95% CI), *P* value	AOR (95% CI), *P* value
**Gender**					
Male	1150 (83.7)	224 (16.3)	1374	1.00	1.00
Female	712 (85.4)	122 (14.6)	834	0.88 (0.69–1.12), 0.294	0.95 (0.73–1.23), 0.682
**Age (years)**					
≤ 20	217 (88.9)	27 (11.1)	244	1.00	1.00
21–40	432 (76.7)	131 (23.3)	563	2.44 (1.56–3.80), < 0.0001	1.73 (1.09–2.76), 0.021
41–60	580 (82.0)	127 (18.0)	707	1.76 (1.13–2.74), 0.013	1.11 (0.70–1.78), 0.652
> 60	633 (91.2)	61 (8.8)	694	0.77 (0.48–1.25), 0.295	0.54 (0.33–0.89), 0.016
**Ethnicity**					
Han	1580 (84.5)	290 (15.5)	1870	1.00	1.00
Tujia	116 (85.3)	20 (14.7)	136	0.94 (0.58–1.53), 0.803	1.27 (0.62–2.59), 0.509
Miao	76 (82.6)	16 (17.4)	92	1.15 (0.66–1.99), 0.627	0.76 (0.41–1.41), 0.384
Kelao	55 (83.3)	11 (16.7)	66	1.09 (0.56–2.11), 0.799	0.99 (0.54–1.82), 0.974
Other minorities	35 (79.5)	9 (20.5)	44	1.40 (0.67–2.95), 0.374	1.01 (0.44–2.31), 0.981
**City (n = 2207)**					
Zunyi	1369 (85.1)	239 (14.9)	1608	1.00	1.00
Tongren	233 (81.5)	53 (18.5)	286	1.30 (0.94–1.81), 0.114	1.44 (0.95–2.20), 0.088
Bijie	177 (83.1)	36 (16.9)	213	1.17 (0.79–1.71), 0.435	1.10 (0.72–1.68), 0.653
Other cities of GZ	66 (80.5)	16 (19.5)	82	1.39 (0.79–2.44), 0.253	1.13 (0.61–2.09), 0.696
Cities outside GZ	16 (88.9)	2 (11.1)	18	0.72 (0.16–3.13), 0.657	0.91 (0.19–4.30), 0.908
**Treatment history**					
New	1479 (91.1)	144 (8.9)	1623	1.00	1.00
Retreated	383 (65.5)	202 (34.5)	585	5.42 (4.25–6.90), < 0.0001	5.17 (4.02–6.63), < 0.0001
**TB type**					
PTB	1127 (82.3)	243 (17.7)	1370	1.00	1.00
PTB & EPTB	735 (87.7)	103 (12.3)	838	0.65 (0.51–0.83), 0.001	0.73 (0.56–0.95), 0.018
Total	1862 (84.3)	346 (15.7)	2208	N/A	N/A

*AOR* adjusted odds ratio, *CI* confidence interval, *EPTB* extrapulmonary TB, *GZ* Guizhou province, *N/A* not applicable, *OR* odds ratio, *PTB* pulmonary TB, *PTB & EPTB* pulmonary TB concurrent with EPTB, *RR-TB* rifampicin-resistant TB, *RS-TB* rifampicin-susceptible TB.

We conducted univariate and multivariate logistic regression analyses of risk variables for association with important TB characteristics such as retreated and RR-TB. As shown in Tables 1 and 2, patients from other cities (ORs between 1.29 and 1.49) had higher odds of having concurrent EPTB compared to those from Zunyi, and retreated patients had the highest odds (OR 5.42 and adjusted odds ratio (AOR) 5.17, *P* < 0.0001) of being RR-TB compared to new patients. Furthermore, patients between 21 and 40 years had a higher likelihood (OR 2.44, *P* < 0.0001; AOR 1.73, *P* = 0.021), while patients over 60 years had a lower likelihood of being RR-TB compared to those 20 years of age and younger. Patients with concurrent EPTB had lower odds of being RR-TB (OR 0.65, *P* = 0.001; AOR 0.73, *P* = 0.018) compared to PTB patients (Table 2).

**Figure 2:**
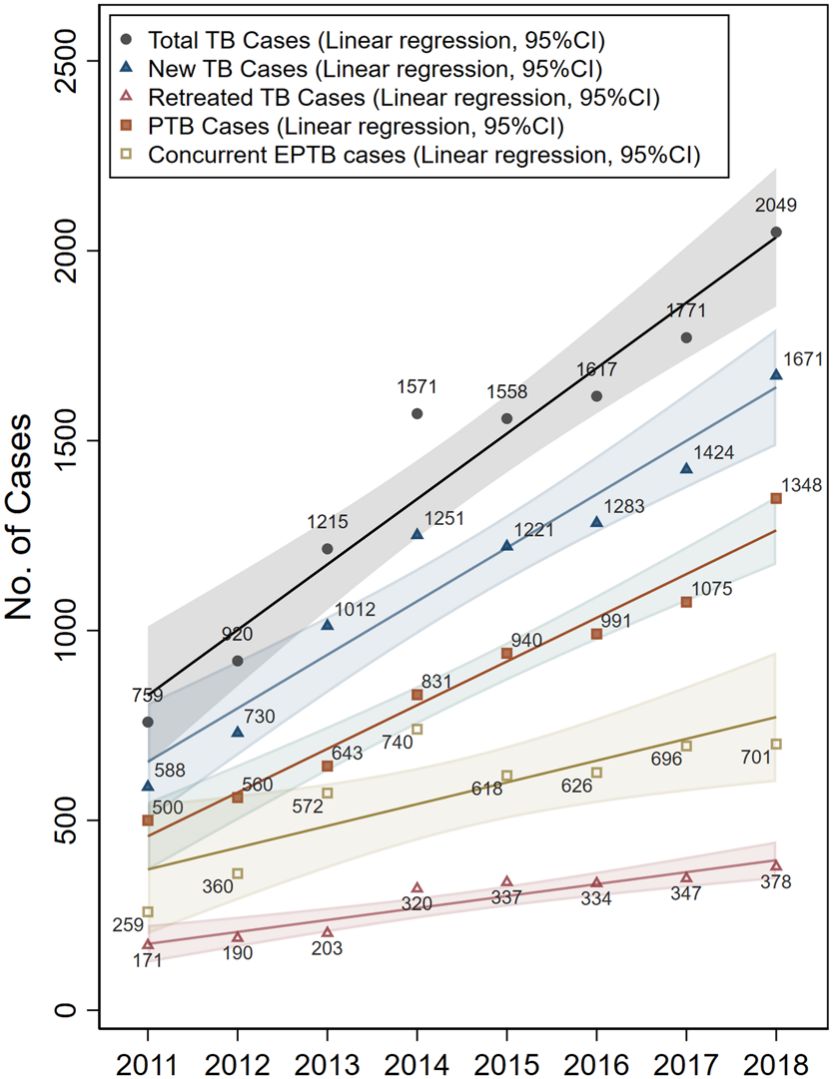
Annual numbers of total, new, retreated, pulmonary, and concurrent extrapulmonary TB cases from 2011 to 2018 with 95% confidence intervals.

To analyze the trends, we compared annual TB cases from 2011 to 2018. During the study period, the number of new TB cases increased 1.84 times, the number of retreated TB cases increased 1.21 times, and the number of concurrent EPTB cases increased 1.71 times (Table 3, Fig. 2). Overall, annual numbers of new, retreated, RR-TB, and concurrent EPTB cases increased from 2011 to 2018 in northern Guizhou Province of China; and the upward trends were statistically significant from 2015 to 2018 (P < 0.05) (Table 3, Fig. 2).

**Table 3:** Annual numbers and percentages of new, retreated, PTB, concurrent EPTB, tested for RR, RS-TB and RR-TB cases from 2011 to 2018.

Year	Total cases	New, n (%)	Retreated, n (%)	Chi-square test for trend, *P* value		PTB, n (%)	PTB & EPTB, n (%)	Chi-square test for trend, *P* value		Cases tested for RR, n (%)	RS-TB, n (%)	RR-TB, n (%)	Chi-square test for trend, *P* value	
				2011–2018	2015–2018			2011–2018	2015–2018				2011–2018	2015–2018
2011	759	588 (77.5)	171 (22.5)	0.2017		500 (65.9)	259 (34.1)	< 0.0001		89 (11.7)	81 (91.0)	8 (9.0)	0.4189	
2012	920	730 (79.3)	190 (20.7)			560 (60.9)	360 (39.1)			24 (2.6)	19 (79.2)	5 (20.8)		
2013	1215	1012 (83.3)	203 (16.7)			643 (52.9)	572 (47.1)			87 (7.2)	70 (80.5)	17 (19.5)		
2014	1571	1251 (79.6)	320 (20.4)			831 (52.9)	740 (47.1)			188 (12.0)	151 (80.3)	37 (19.7)		
2015	1558	1221 (78.4)	337 (21.6)		0.0122	940 (60.3)	618 (39.7)		0.0012	242 (15.5)	211 (87.2)	31 (12.8)		0.0486
2016	1617	1283 (79.3)	334 (20.7)			991 (61.3)	626 (38.7)			343 (21.2)	295 (86.0)	48 (14.0)		
2017	1771	1424 (80.4)	347 (19.6)			1075 (60.7)	696 (39.3)			537 (30.3)	459 (85.5)	78 (14.5)		
2018	2049	1671 (81.6)	378 (18.4)			1348 (65.8)	701 (34.2)			698 (34.1)	576 (82.5)	122 (17.5)		

*EPTB* extrapulmonary TB, *PTB* pulmonary TB, *PTB & EPTB* pulmonary TB concurrent with EPTB, *RR* rifampicin resistance, *RR-TB* rifampicin-resistant TB, *RS-TB* rifampicin-susceptible TB.

## Discussion

Our study showed the overall upward trends in new, retreated, RR-TB and concurrent EPTB cases from 2011 to 2018. However, results from analyzing annual proportions of these cases showed a fluctuant period (2011 to 2015) and a steadily changing period (2015 to 2018), and that 15.7% of tested TB cases were RR-TB which was consistent with our previous studies^14–16^. During the first period (2011 to 2015), the percentage of retreated TB cases declined initially and then increased, while those of new TB, RR-TB and concurrent EPTB cases increased initially and then declined. During the second period (2015 to 2018), the percentage of retreated TB cases declined continuously, but percentages of new and RR-TB cases increased, which was similar to a rising trend in RR-TB cases from 2005 to 2015 observed by Beijing Chest Hospital^17^, from 2004 to 2019 in elderly TB patients in Shandong Province^18^, and from 2010 to 2017 observed in Chongqing City^19^. The upward trend in RR-TB cases would certainly hold back TB control efforts in northern Guizhou Province.

Three past achievements of our department which enhanced the brand awareness of the hospital might have indirectly influenced the fluctuation in percentages of RR-TB and concurrent EPTB cases from 2011 to 2015. First, our department was designated by the provincial government as the “Key Clinical Department of Guizhou Province” in 2011. Second, our department received grants from the “Global Fund” and “National Science and Technology Major Projects” to support patients with MDR/RR-TB to receive standardized diagnosis and treatment between 2011 and 2013. Third, our hospital was designated by the provincial government in 2014 as a specialized hospital for MDR-TB patients. Consequently, more patients with RR-TB, concurrent EPTB, and complex diseases were referred to our hospital from 2011 to 2015. These effects gradually stabilized over the time; therefore, we think that the period from 2015 to 2018 would reflect more practically the current TB trends in northern Guizhou Province of China.

The proportion of laboratory confirmed TB cases had increased from 2011 to 2018, especially in recent years with the addition of molecular tests, indicating a continuous improvement in diagnosis of TB. The higher proportion of laboratory confirmed TB cases also provided more isolates for DST, resulting in improved treatment outcomes. The percentage of retreated TB cases is an important indicator of treatment outcomes. Data from the Fifth National TB Epidemiological Survey (2010) showed that percentages of retreated TB cases in both urban and rural areas of western China were higher than those in eastern and central regions of China^20^. However, the percentage of retreated TB cases from this study (20%) was slightly lower than that of China (21%). A steady downward trend in the annual percentage of retreated TB cases from 21.6% (2015) to 18.4% (2018) showed improved treatment success rates in the region and indicated that the increasing number of total TB cases was mainly caused by primary transmission rather than treatment failure.

Retreatment, RR-TB and EPTB are negative factors affecting the treatment outcome and prognosis of patients. Previous treatment history has been a well-known risk factor associated with drug-resistant TB including RR-TB^21–23^. Our result that patients between 21 and 40 years of age had higher odds of being RR-TB compared to those 20 years and younger was consistent with past studies^21,24,25^. However, more studies would be needed to verify our result that patients over 60 years had the lowest likelihood of being RR-TB compared to those in other age groups, since the difference was not statistically significant.

Previous studies considered ≥ 45 years of age, female gender, HIV-positive, and the end-stage rental disease as risk factors for EPTB^26–28^. However, our study showed that female gender and ≤ 20 years of age were more likely to have concurrent EPTB, which were consistent with reports from high-burden countries such as Nepal, China and Pakistan^29–31^. Our result that patients with concurrent EPTB had lower odds of being RR-TB was consistent with past studies^32^. One possible explanation is that most concurrent EPTB cases in our study were new cases which were less likely to be associated with RR-TB. Among different ethnic groups, patients from the Miao ethnic group had the highest likelihood of having EPTB in comparison with the Han majority, which might be better explained by geographic, socioeconomic, and systemic factors. Our study also showed that retreated and RR-TB cases were correlated with each other but they were less likely to be associated with concurrent EPTB.

Our study has a few potential limitations. First, hospitalized patients tend to be more serious and patients attending the University Hospital may be more prestigious or wealthy than outpatients, therefore, actual proportions of retreated, RR-TB and concurrent EPTB cases in northern Guizhou Province might be lower than what we reported here, but trends in the region should remain the same. Similarly, Beijing Chest Hospital reported the RR-TB proportion of 30.5% in 2012^33^, which was higher than the national average. Second, due to the limited scope of the assay, the GeneXpert MTB/RIF cannot detect uncommon mutations in the *rpoB* gene such as the Ile572Phe (Ile491Phe in the *M. tb* numbering system) mutation, especially in geographical locations where this mutation is frequent^34,35^. Therefore, the actual proportion of RR-TB could be slightly higher than what we observed in this study. Third, the findings of this study were based only on the data from one major hospital in northern Guizhou province and should not be used to represent the entire Guizhou province because of limitations related to access to the data for the entire province.

In conclusion, our findings show upward trends in new, RR-TB, and concurrent EPTB cases in northern Guizhou Province of China, indicating that trends in RR-TB and concurrent EPTB could go up or down sporadically in some regions of high TB burden countries, even though overall trends in TB cases continued to decline for the past 20 years in most of the 30 high TB burden countries. To achieve the 2030 targets of the End TB Strategy to reduce the number of TB deaths by 90% and the TB incidence rate by 80% in comparison with levels in 2015, it will be important to enhance TB control programs in regions of the high TB burden countries with upward trends in RR-TB and concurrent EPTB cases. The TB experts who work with governments and international organizations should make specific recommendations to help high TB burden countries to decide which policies they should implement according to their own missions and capabilities.
